# Hyperoxia-induced methylation decreases *RUNX3* in a newborn rat model of bronchopulmonary dysplasia

**DOI:** 10.1186/s12931-015-0239-x

**Published:** 2015-06-24

**Authors:** Yuting Zhu, Jianhua Fu, Haiping Yang, Yuqing Pan, Li Yao, Xindong Xue

**Affiliations:** Department of Pediatrics, Shengjing Hospital of China Medical University, Shenyang, 110004 China

**Keywords:** DNA methylation, histone methylation, *RUNX3*, alveolar development, bronchopulmonary dysplasia, EZH2

## Abstract

**Background:**

Bronchopulmonary dysplasia (BPD) in premature infants is a predominantly secondary occurrence to intrauterine inflammation/infection and postpartum mechanical ventilation; in recent years, an association with epigenetics has also been found. DNA methylation, catalyzed by DNA methyl transferases (DNMTs), and tri-methylation of lysine 27 on histone H3 (H3K27me3), mediated by the methyltransferase, Enhancer of Zeste Homolog 2 (EZH2), are some of the most commonly found modifications in epigenetics. Runt-related transcription factor 3 (*RUNX3*) is associated with pulmonary epithelial and vascular development and regulates expression at the post-transcriptional level by DNA methylation through DNMT1 or DNMT3b. However, the involvements of these epigenetic factors in the occurrence of BPD are, as yet, unclear.

**Methods:**

Newborn rats were randomly assigned to a model, hyperoxia (85 % O_2_) or control, normoxia group (21 % O_2_). Lung tissues and alveolar type 2 (AT2) epithelial cells were collected between 1–14 days. The expression of DNMTs, and EZH2 was detected by immunohistochemistry, Western blot and real-time PCR. The percentage of DNA methylation and H3K27me3 levels in the *RUNX3* promoter region was measured by bisulfite sequencing PCR and chromatin immunoprecipitation assay. RUNX3 protein and mRNA expression in AT2 cells was also measured after inhibition using the DNA methylation inhibitor, 5-Aza-2′-deoxycytidine, the H3K27me3 inhibitor, JMJD3, and the EZH2 inhibitor, DZNep.

**Results:**

Compared with the control group, RUNX3 protein was downregulated and DNMT3b and EZH2 were highly expressed in lung tissues and AT2 cells of the model group (*P* < 0.05), while high DNA methylation and H3K27me3 modifications were present in the *RUNX3* promoter region, in lung tissues of the model group (*P* < 0.05). Following hyperoxia in the model group, JMJD3 and DZNep significantly reversed the hyperoxia-induced down-regulation of RUNX3 expression in AT2 cells (*P* < 0.05), more so than 5-Aza-2′-deoxycytidine (*P* < 0.05).

**Conclusions:**

1) DNA methylation and H3K27 trimethylation are present in the BPD model; 2) RUNX3 down-regulation is attributed to both DNMT3b-catalyzed DNA methylation and EZH2-catalyzed histone methylation.

## Background

Bronchopulmonary dysplasia (BPD) is a common, chronic lung disease of infants, frequently seen in premature newborns with a fetal age < 30 weeks and a birth weight < 1,500 g. The occurrence of BPD is mostly secondary to intrauterine inflammation/infection or postpartum mechanical ventilation, oxygen support, and changes in other environmental factors. The main pathological changes that characterize BPD are alveolar structure simplification and pulmonary capillary dysplasia [1]. In recent years, a large number of studies have revealed that some genes, including those for vascular endothelial growth factor (VEGF) [2], interleukin 1-beta (IL-1β) [3] and mucin 1 (MUC1) [4], participate in the pulmonary developmental disorder process of BPD by regulating alveolar formation.

Runt-related transcription factor 3 (*RUNX3*) is a member of the RUNX family and participates in normal physiological, as well as pathological, processes of the immune system [5], in tumor formation [6] and in other disorders. *RUNX3* is also associated with pulmonary epithelial and vascular development [7]. Previous studies have shown that RUNX3 was expressed in mouse pulmonary epithelium at E15.5 [8], while *RUNX3* knock-out caused pulmonary epithelial hyperplasia [8] and pulmonary vascularization disorder [7], similar to the pathological changes seen in BPD [1]. *RUNX3* often regulates expression at the post-transcriptional level by DNA methylation [9]. However, the mechanisms behind RUNX3 down-regulation and any potential regulators of abnormal RUNX3 expression in a BPD model have, as yet, to be defined.

The silencing of RUNX3 expression is associated with the tri-methylation of lysine 27 on histone H3 (H3K27me3), an epigenetic marker, and is mediated by the methyltransferase, Enhancer of Zeste Homolog 2 (EZH2) [10, 11] and demethyltransferase, JMJD3/UTX [12], to reduce transcription [13]. Fujii et al. [14] found that *EZH2* knock-out reduced H3K27me3-binding RUNX3 levels and thus up-regulated *RUNX3* mRNA levels.

DNA methyl transferases (DNMTs) catalyze DNA methylation, which leads to the silencing of gene expression. Common DNMTs include DNMT1, which maintains and regulates DNA methylation, and DNMT3a/b, which establishes *de novo* methylation [15, 16]. DNMT1 was thought to be the major contributor of *RUNX3* DNA methylation, but DNMT3b has also been found to have a role [17]. Additionally, Deng et al. [10] found that the inhibition of DNMT3b expression caused the upregulation of RUNX3 expression in a colorectal cancer cell line.

Numerous studies have suggested that BPD is a genetically susceptible disease. Studies of twins have shown that the BPD status of one twin was a significant predictor of BPD in the second twin [18], and that the incidence of BPD in homozygotic twins was significantly greater than that of dizygotic twins [19]. Subsequently, many scholars have reported abnormalities of histone acetylase activity and the chromatin remodeling pathway in BPD patients, and believe that epigenetics is a causal factor in the occurrence and development of BPD [20–23]. However, whether two common epigenetic modifications–DNA methylation and H3K27me3–are associated with BPD [24], and whether, by regulating target genes, they participate in the pulmonary developmental disorder processes of BPD is unclear. Consequently, this study aimed to identify the presence or absence of DNA methylation and H3K27me3 in BPD, and to highlight any correlation between RUNX3 down-regulation and DNA methylation or H3K27me3 in BPD at the epigenetic level.

## Experimental methods

### Animal model and tissue specimens

A newborn rat model of BPD, established by our research group as previously described, was used [25]. Two hundred newborn, Sprague–Dawley (SD) rats were randomly divided into a model (exposure to hyperoxia [85 % O_2_] from day of birth) or control group (exposure to normoxia [21 % O_2_]). To avoid O_2_ toxicity, maternal rats within the model and control groups were switched once every 24 h. Rats were given *ad libitum* access to water and food. At 1, 7, 10 and 14 days after the start of exposure to hyperoxia or normoxia, eight newborn rats from each model or control group were anesthetized by intraperitoneal injection with 5 % chloral hydrate, and whole lungs collected aseptically by chest opening. The left lungs were fixed in paraformaldehyde (PFA) for subsequent immunohistochemical staining, the right upper lung lobes were used for real-time PCR analysis, and the right lower lung lobes for Western blots. All specimens were snap-frozen in liquid nitrogen and stored at −80 °C until use. Mature SD rats with a body weight of 220–250 g were purchased from the Department of Animals, Experimental Center, Shengjing Hospital of China Medical University (Shenyang, China). All animal experiments were approved and supervised by the Ethics Committee of Animals, China Medical University.

### AT2 cell isolation and purification

The above BPD animal model was employed. At 0, 1, 7, 10 and 14 days after the start of normoxia or hyperoxia, alveolar type 2 (AT2) epithelial cells of newborn rats were isolated from the control or model groups, respectively, for primary culture. As previously described [26, 27], tracheal intubation was performed on anesthetized rats to maintain lung ventilation and to conduct the subsequent lavage. Two cold buffer solutions and an albumin emulsion were used for cardiopulmonary and trachea cannula lavage to remove blood and macrophages from lung tissues. AT2 cells were isolated by mechanical separation and trypsinization of lung tissue, followed by filtration with a nylon mesh. A first-round purification was conducted based on differential cell adherence, and a second-round purification was performed using a lamellar body cell membrane antibody p180 (Abcam, Cambridge, MA, USA). After storage in liquid nitrogen, AT2 cells of the model and control groups, isolated at 1, 7, 10 and 14 days, were transferred to a −80 °C freezer and then subsequently used for real-time PCR, Western blot, bisulfite sequencing PCR (BSP) analysis and chromatin immunoprecipitation (ChIP) assay. Purified AT2 cells, extracted immediately after birth (0 day), were used for inhibitor treatments as described below.

### Cell treatment

Purified AT2 cells extracted immediately after birth (0 day) were plated out into 6-well plates and then cultured in environments of differing oxygen concentrations: a control (21 % O_2_) or model (85 % O_2_) group. Each group of cells was cultured in the presence of various concentrations of 5-Aza-2′-deoxycytidine (5-Aza-CdR, Abcam; 2.5, 5 or 10 μmol/L), Jumonji domain containing 3 (Jmjd3, Abcam; 10, 20 or 40 μg/L) or 3-Deazaneplanocin A (DZNep, Selleckchem, Shanghai, China; 1, 2.5 or 5 μmol/L) for 48 h, or treated with 5 μmol/L 5-Aza-CdR, 20 μg/L Jmjd3 or 1 μmol/L DZNep for 24, 48 or 72 h. Inhibitors and media were changed every 24 h until cells were collected. Cells of the negative control and model groups were cultured without inhibitors. After storage in liquid nitrogen, cells were transferred to a −80 °C freezer and then subsequently used for real-time PCR and Western blotting.

### Immunohistochemistry

PFA-fixed lung tissues were dehydrated, vitrified, embedded in paraffin, fixed and cut into 4 μm-thick sections, which were then fixed in a 60 °C oven for 4 h. Sections were dewaxed with dimethyl benzene, hydrated with gradient alcohol according to manufacturer’s instructions (immunohistochemistry kit; MXB, Fujian, China), and treated with 3 % H_2_O_2_ to block endogenous peroxidase activity. Treated sections were placed into an EDTA-Tris buffer solution and microwaved for 20 min, blocked with serum, and incubated overnight at 4 °C with various antibodies: rabbit polyclonal anti-RUNX3, 1:200 (Abcam); rabbit polyclonal anti-DNA methyltransferase 3b, 1:500 (DNMT3b, Abcam); mouse monoclonal anti-H3K27me3, 1:1000 (Abcam); rabbit polyclonal anti-DNA methyltransferase 1, 1:50 (DNMT1; Santa Cruz Biotechnology, Dallas, TX, USA); or mouse monoclonal anti-Enhancer of Zeste Homolog 2, 1:200 (EZH2; Becton, Dickinson and Co, Franklin Lakes, NJ, USA). After sequential incubation with biotin-labeled secondary antibodies and streptavidin-peroxidase, sections were developed with 3,3′-diaminobenzidine (DAB), dehydrated and mounted. A laser scanning confocal microscope (MTC-600, Bio-Rad, Hercules, CA, USA) was used for image acquisition, and the deposition of brown particles in the cytoplasm/nucleus indicated a positive result. Cells displaying brown particles in the cytoplasm/nucleus were counted within the same sized area as viewed under the microscope.

### Western blot

An EpiQuik Nuclear Extraction Kit (Epigentek Group Inc, New York, NY, USA) was used to extract total nuclear protein from lung tissues and AT2 cells after exposure to normoxia or hyperoxia, and AT2 cells after inhibitor treatment. After SDS polyacrylamide gel electrophoresis (SDS-PAGE), separated proteins were transferred onto a polyvinylidene fluoride (PVDF) membrane, and the membrane was then blocked with 10 % skimmed milk and incubated overnight with various antibodies: (anti-RUNX3, 1:800; anti-DNMT3B, 1:1000; anti-H3K27me3, 1:3000; anti-DNMT1, 1:200; anti-EZH2, 1:1000; anti-GAPDH, 1:15,000 [all Santa Cruz Biotechnology]). The membrane was then incubated in secondary antibody for 4 h, washed in 10 mM Tris/HCl, 150 mM NaCl, and 0.05 % Tween 20, pH 7.5 (TBST) buffer three times, developed with enhanced chemiluminescence substrate (ECL kit; Santa Cruz Biotechnology) and exposed to X-ray film. All bands on X-ray film were scanned using ChemiImager 5500 V2.03 software. Integrated density values were computed using an image analysis system (Fluor Chen 2.0; Bio-Rad, Hercules, CA, USA) and standardized to GAPDH.

### Real-time PCR

Total mRNA was extracted from right upper lung lobe tissues and AT2 cells after exposure to normoxia or hyperoxia, and from AT2 cells after inhibitor treatment. Briefly, Trizol, chloroform and isopropyl alcohol were added and samples subjected to reverse transcription and real-time PCR (Life Technologies, Carlsbad, CA, USA) according to kit instructions. Reverse transcription conditions were: 65 °C, 5 min; 37 °C, 2 min; 37 °C, 50 min; and 70 °C, 15 min. PCR conditions were 50 °C, 2 min; 95 °C, 2 min; 95 °C, 15 s; and 60 °C, 1 min for 40 cycles. Primer sequences of different indicators are shown in Table 1, and model data were standardized to GAPDH.

**Table 1:** Real-time PCR primers for genes

Target gene		5′ → 3′	Product
dnmt1	Sense	CATGGTGCTGAAGCTCACAC	176 bp
	Antisense	GGGCAAACACGTGTAGAGGT	
dnmt3b	Sense	TTCTCATGATGCCAAAGCTC	118BP
	Antisense	GAGGTTCTTTGCCTCTCCAG	
ezh2	Sense	TTCGTTTTGCTAATCATTCAGTAA	162 bp
	Antisense	CCACATACTTCAGGGCATCA	
runx3	Sense	GAAGATAGAGGACCAGACCAAAG	194 bp
	Antisense	GGAAGGAGCGATCAAACTG	
gapdh	Sense	AGACAGCCGCATCTTCTTGT	207 bp
	Antisense	CTTGCCGTGGGTAGAGTCAT	

### Bisulfite sequencing PCR (BSP)

After 7 and 14 days, the genomic DNA of AT2 cells in model and control groups was extracted using a blood/cell/tissue genomic DNA extraction kit (Tiangen Biotech, Beijing, China). After elution with 70 °C pre-heated, sterile water, DNA was collected. Thereafter, DNA specimens were treated with a DNA methylation kit (Millipore, Billerica, MA, USA) and amplified. PCR amplification conditions were 95 °C, 5 min; 95 °C, 10 s; 50 °C, 20 s; 72 °C, 30 s; 35 cycles; 4 °C, 5 min. Forward primer: 1: 5′-GGATTAAGGTTGAGAAGATGATGG-3′, reverse primer 1: 5′-ACCACCCTATTCCTACCCACTC-3′; forward primer 2: 5′-TTAGAAGGGCGTTTAGGAGA-3′, reverse primer 2: 5′-AACTCTAACGATCCTCATCC-3′. Amplicons were checked on agarose gel and sequenced using BiQ Analyzer.

### Chromatin Immunoprecipitation (ChIP) Assay

Chromatin co-immunoprecipitation was performed according to manufacturer’s instructions (EZ-ChIP kit; Millipore). In brief, 1 % (final concentration) formaldehyde was used to treat AT2 cells isolated from model and control groups at 14 days; after protein-DNA cross-linking, genomic DNA was ultrasonically sheared into lengths of 200–1000 bp. Non-specific binding proteins and DNA were pre-removed with Protein G agarose and an antibody-free control sample was put aside. Normal mouse IgG (IgG group), anti-H3K27me3 (target gene group 1; 5 μL [Abcam]) or anti-EZH2 (target gene group 2; 2 μL [Millipore]) and Protein G agarose were added to samples and incubated overnight. Samples were washed according to instructions, eluted with elution buffer to remove protein/DNA complexes, cross-linked, purified step-by-step, and then amplified. Amplification conditions were the same as for real-time PCR, and primer sequences used are shown in Table 2. The model results are expressed as 2^-CT (Target gene group-Input group-IgG group)^.

**Table 2:** CHIP-Real-time PCR primers for runx3

Gene locus		5′ → 3′	Product
1	Sense	CTGTGGCTAAGAGGGTGAC	265 bp
	Antisense	ACAAGGCTGAAGATGACG	
2	Sense	TCTCCACCTCAGAACGC	192 bp
	Antisense	ACAAGGCTGAAGATGACG	
3	Sense	CGCATCCACTTCCACTACAC	180 bp
	Antisense	TTCCTGCCCACTCAAAA	

### Statistical analysis

SPSS17.0 software (manufacture, city, country) was used for statistical analysis. Inter-group comparisons were made using *t* tests, multiple group comparisons made using one-way analysis of variance (ANOVA), and correlation analysis made using Pearson’s test. All data are presented as mean (χ) ± standard deviation (SD). *P* < 0.05 suggests a statistically significant difference.

## Results

### RUNX3 expression in lung tissues and AT2 cells following hyperoxia

**Fig. 1:**
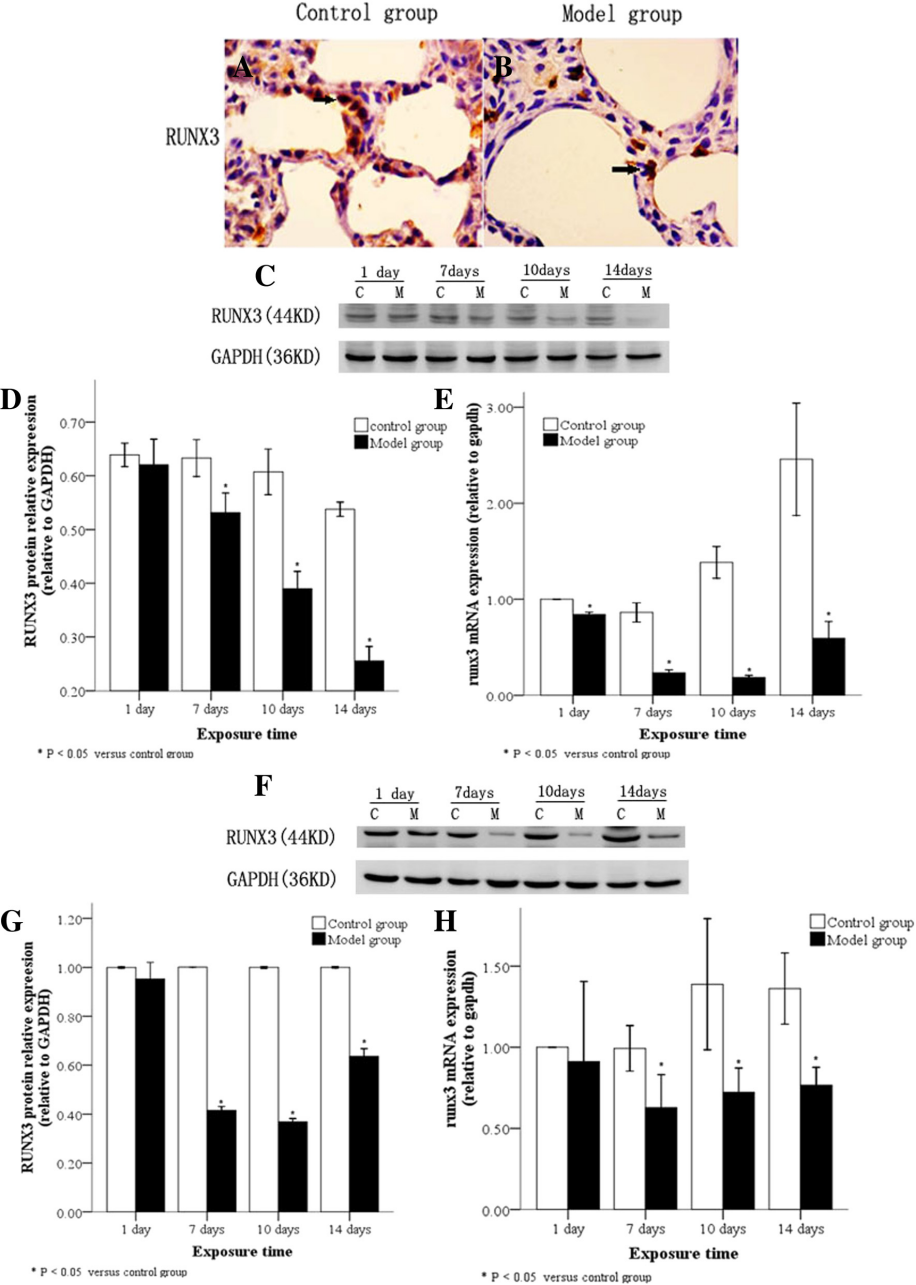
RUNX3 expression in lung tissues and AT2 cells following hyperoxia. RUNX3 protein localization in lung tissues by immunohistochemistry showing RUNX3 protein expression in both the nucleus and cytoplasm of alveolar epithelial cells in control (**a**) and model groups (**b**). The arrow indicates positive cells. (×400). Western blot of RUNX3 protein (**c** and **d**), and real-time PCR of RUNX3 mRNA (**e**) showing low expression in lung tissues of the model group after 7 d hyperoxia. Low expression of RUNX3 protein (**f** and **g**) and mRNA (**h**) in AT2 cells from the model group after 7 d hyperoxia. C: Control group, E: model group, *P < 0.05 vs. control group, n = 4

We studied RUNX3 protein localization by immunohistochemical staining of lung tissues from both control and BPD model groups exposed to hyperoxia over time; RUNX3 protein expression was found in the nucleus and cytoplasm of alveolar epithelial cells in lung sections of both the control and model groups (Fig. 1a, b). We also examined RUNX3 protein and mRNA levels in lung tissue and AT2 cells isolated from these groups. Compared with the control group, RUNX3 protein and mRNA expression in lung tissues and AT2 cells from the model group was significantly decreased by ≥ 7 days of hyperoxia (Fig. 1; *P* < 0.05).

### DNMT and histone methyltransferase EZH2 localization in lung tissues following hyperoxia

**Fig. 2:**
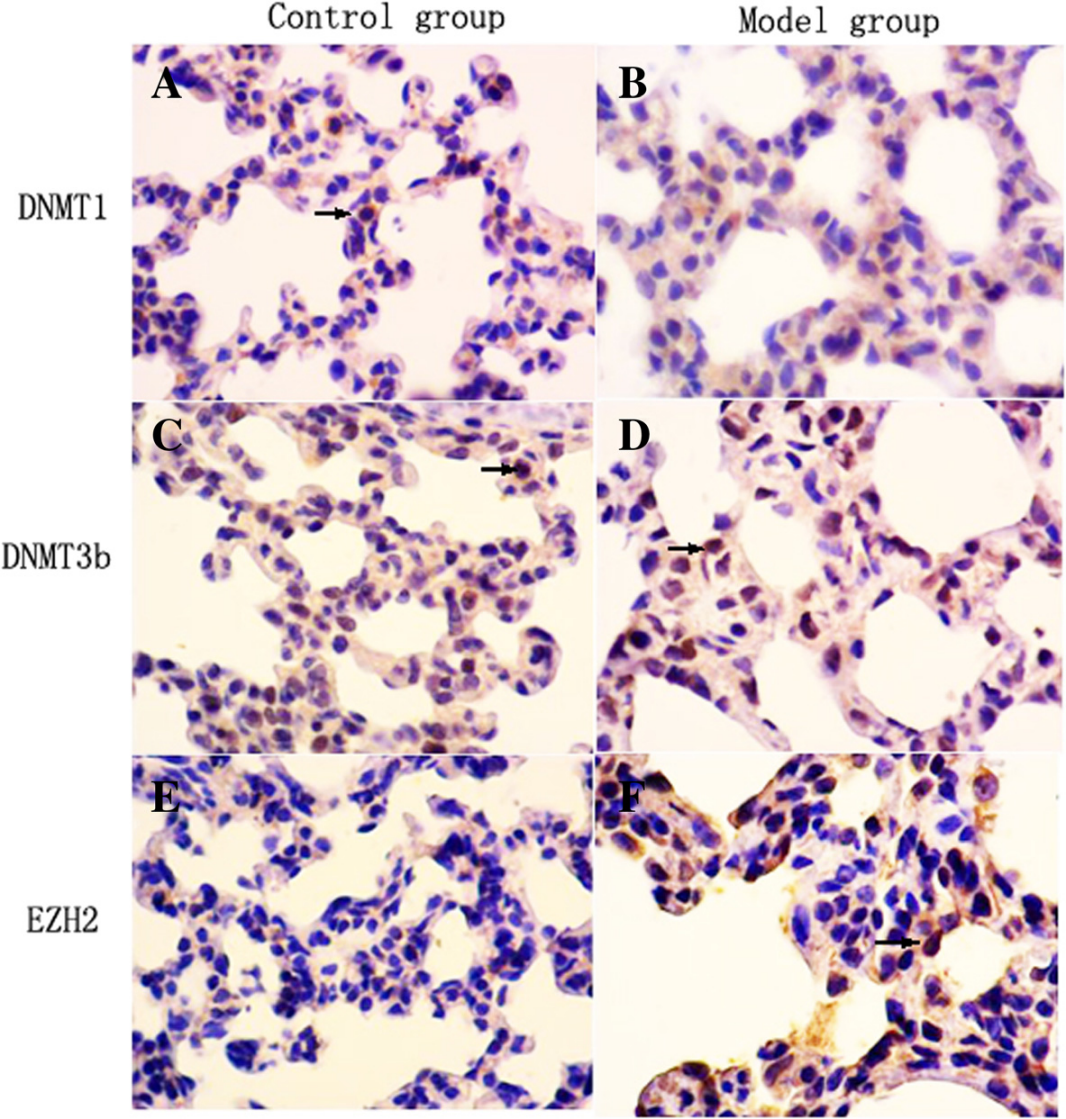
DNMTs and EZH2 protein localization in lung tissues following hyperoxia. DNMTs and EZH2 protein localization in lung tissues by immunohistochemistry. After 10 day of hyperoxia, DNMT1 protein was expressed in pulmonary alveolar epithelial cells in the control group (**a**), but, in the model group, DNMT1 protein was barely detectable (**b**). In both the control (**c**) and model groups (**d**), DNMT3b protein was expressed in alveolar epithelial and mesenchymal cells. In the control group, EZH2 protein was poorly expressed (**e**). In the model group, EZH2 protein was expressed in alveolar epithelial cells (**f**). The arrow points to positive cells. (×400)

The localization of DNMTs and EZH2 proteins in lung tissues of animals exposed to hyperoxia or normoxia was examined by immunohistochemistry. After 10 day of hyperoxia, DNMT1 protein was expressed in both the nucleus and cytoplasm of pulmonary epithelial cells in the control group, but was poorly expressed in the model group (Fig. 2a, b). In both the model and control groups, DNMT3b protein was expressed in the nucleus of alveolar epithelial and mesenchymal cells (Fig. 2c, d). EZH2 protein was expressed in both the nucleus and cytoplasm of alveolar epithelial cells in the model group, but expression was greatly decreased in the control group (Fig. 2e, f).

### DNMT expression levels in lung tissues and A2 cells following hyperoxia

**Fig. 3:**
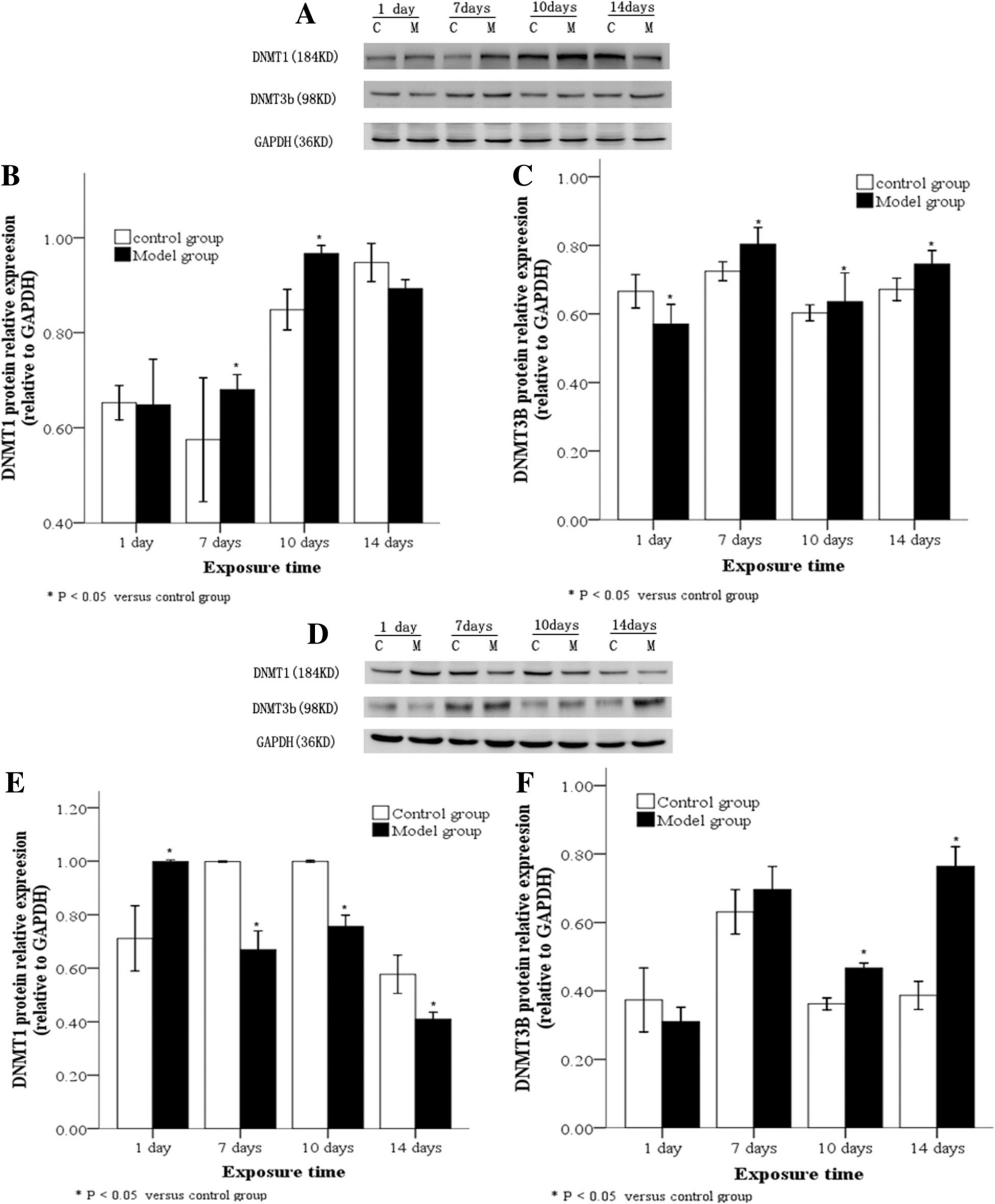
DNMT protein levels in lung tissues and AT2 cells following hyperoxia. Western blot (**a**) showing DNMT1 protein was highly expressed in lung tissues in the model group after between 7–10 day of hyperoxia, (**b**) and DNMT3b was highly expressed in the lower lobe of right lung tissues after 7 days of hyperoxia (**c**). A Western blot of DNMT1 and DNMT3b protein expression in AT2 cells from lung tissue (**d**) showed DNMT1 protein was poorly expressed in AT2 cells from the model group after 7 days of hyperoxia (**e**) while DNMT3b protein was highly expressed in AT2 cells after 10 day of hyperoxia. (**f**). C: Control group, E: Model group, *P < 0.05 vs. control group, n = 4

DNMTs protein levels in lung tissues of animals exposed to hyperoxia or normoxia were also examined by Western blot. Compared with the control group, DNMT1 protein in lung tissues of the model group significantly increased by ≥ 7 days, reached a peak at 10 day, and thereafter decreased (Fig. 3a, b; *P* < 0.05), while DNMT3b protein demonstrated a persistently high expression level after 7 days (Fig. 3a, c; *P* < 0.05). Subsequently, the expression of DNMT1 and DNMT3b in AT2 cells extracted from lung tissues was detected at different time points: Compared with the control group, DNMT1 protein in AT2 cells isolated from the model group rapidly decreased by ≥ 7 days, and demonstrated a persistently and significantly low expression level (Fig. 3d, e; *P* < 0.05), while DNMT3b protein increased by ≥ 10 day and demonstrated a significantly high expression level (Fig. 3d, f; *P* < 0.05).

**Fig. 4:**
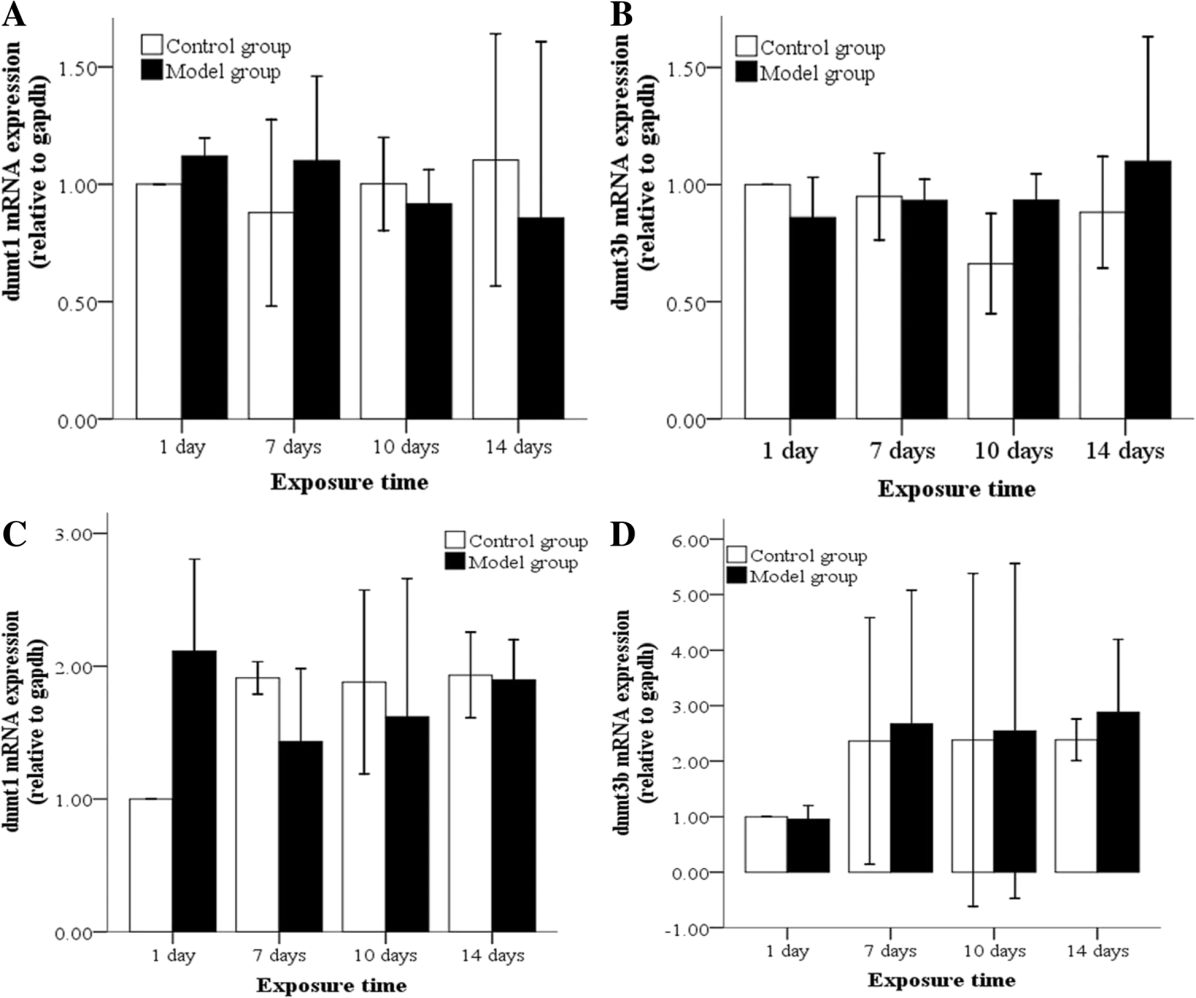
DNMT mRNA levels in lung tissues and AT2 cells following hyperoxia. In the model group, DNMT1 mRNA, as determined by PCR, was poorly (**a**) expressed while DNMT3b mRNA was highly expressed in lung tissues after 10 day of hyperoxia (**b**). After 7 days, DNMT1 mRNA was poorly expressed in AT2 cells of model group (**c**). In both the model and control groups, DNMT3b mRNA was expressed in AT2 cells (**d**), but the difference was not statistically significant after 7 days. *P < 0.05 vs. control group, n = 4

DNMTs mRNA levels in lung tissues and AT2 cells isolated from animals exposed to hyperoxia or normoxia were also examined by real-time PCR. In the model group, DNMT1 mRNA was poorly expressed while DNMT3b mRNA was highly expressed in lung tissues after 10 day of hyperoxia compared to the control group (Fig. 4a, b). After 7 days of hyperoxia, DNMT1 mRNA was poorly expressed in AT2 cells of hyperoxia group (Fig. 4c). For both the model and control groups, DNMT3b mRNA was equally expressed in AT2 cells after 1 day of hyperoxia compared to the control group (Fig. 4d). DNMT1 and DNMT3b mRNA levels in lung tissues and AT2 cells showed similar changes to protein, but the differences between groups were not statistically significant (Fig. 4).

### EZH2 expression levels in lung tissues and A2 cells following hyperoxia

**Fig. 5:**
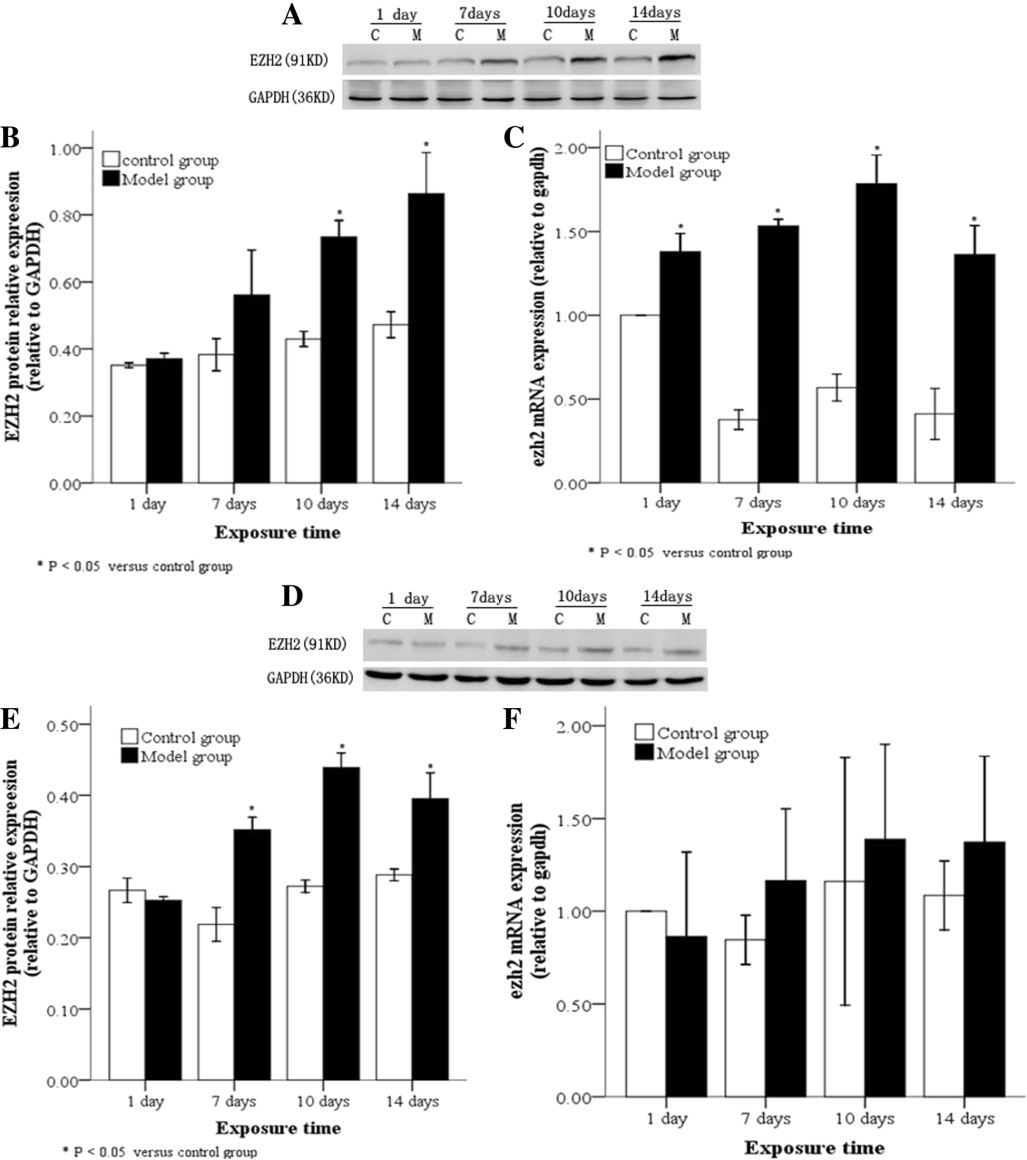
EZH2 protein and mRNA levels in lung tissues and AT2 cells following hyperoxia. In the model group, EZH2 protein, as determined by Western blot, (**a** and **b**) and mRNA, as determined by real time-PCR (**c**), were highly expressed in lung tissues after 10 day of hyperoxia, while EZH2 protein (**d** and **e**) and mRNA (**f**) were highly expressed in AT2 cells after 7 days of hyperoxia. C: Control group, E: Model group, *P < 0.05 vs. control group, n = 4

EZH2 protein levels in lung tissues and AT2 cells of animals exposed to hyperoxia or normoxia were also examined by Western blot. Compared with the control group, EZH2 protein in lung tissues and AT2 cells in the model group was significantly increased at ≥ 10 day of hyperoxia (Fig. 5a, b; *P* < 0.05) and at ≥ 7 days (Fig. 5d, e; *P* < 0.05), respectively. Real-time PCR results revealed that EZH2 mRNA levels in lung tissues were persistently and significantly high from 10 day onwards (Fig. 5c; *P* < 0.05), while they were increased in AT2 cells at ≥ 7 days, but not significantly (Fig. 5f).

### Correlation between RUNX3 expression and DNMTs or EZH2 in AT2 cells

**Fig. 6:**
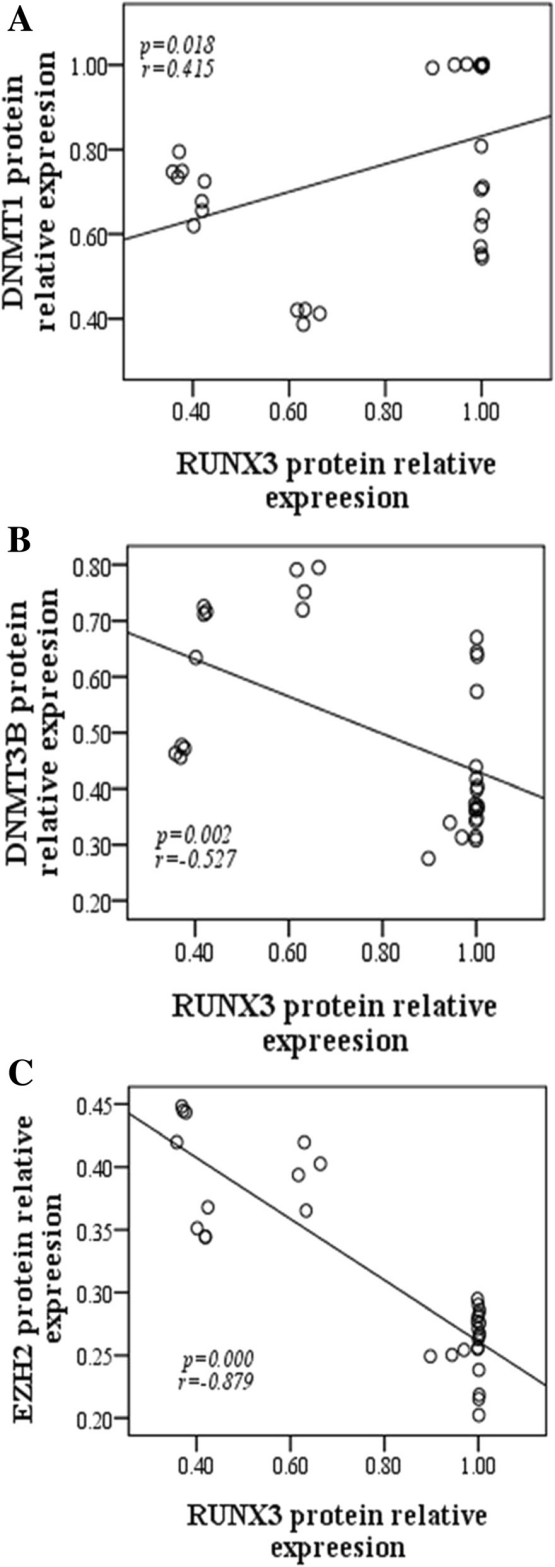
Analysis of the correlation between RUNX3 and DNMTs or EZH2 protein expression in AT2 cells. RUNX3 protein expression correlated positively with DNMT1 protein expression (**a**), but negatively with DNMT3b (**b**) and EZH2 (**c**) protein expression in AT2 cells. *P < 0.05, n = 4

We next carried out correlation analyses between RUNX3 and DNMTs or EZH2 protein expression. DNMT1 protein was positively correlated with RUNX3 protein (Fig. 6a; *r*_1_ = 0.415, *P*_1_ = 0.018), while DNMT3B and EZH2 both showed a significant negative correlation with RUNX3 protein (Fig. 6b, c; *r*_1_ = −0.527, *P*_1_ = 0.002; *r*_2_ = −0.879, *P*_2_ = 0.000, respectively).

### DNA methylation in the *RUNX3* promoter region

**Fig. 7:**
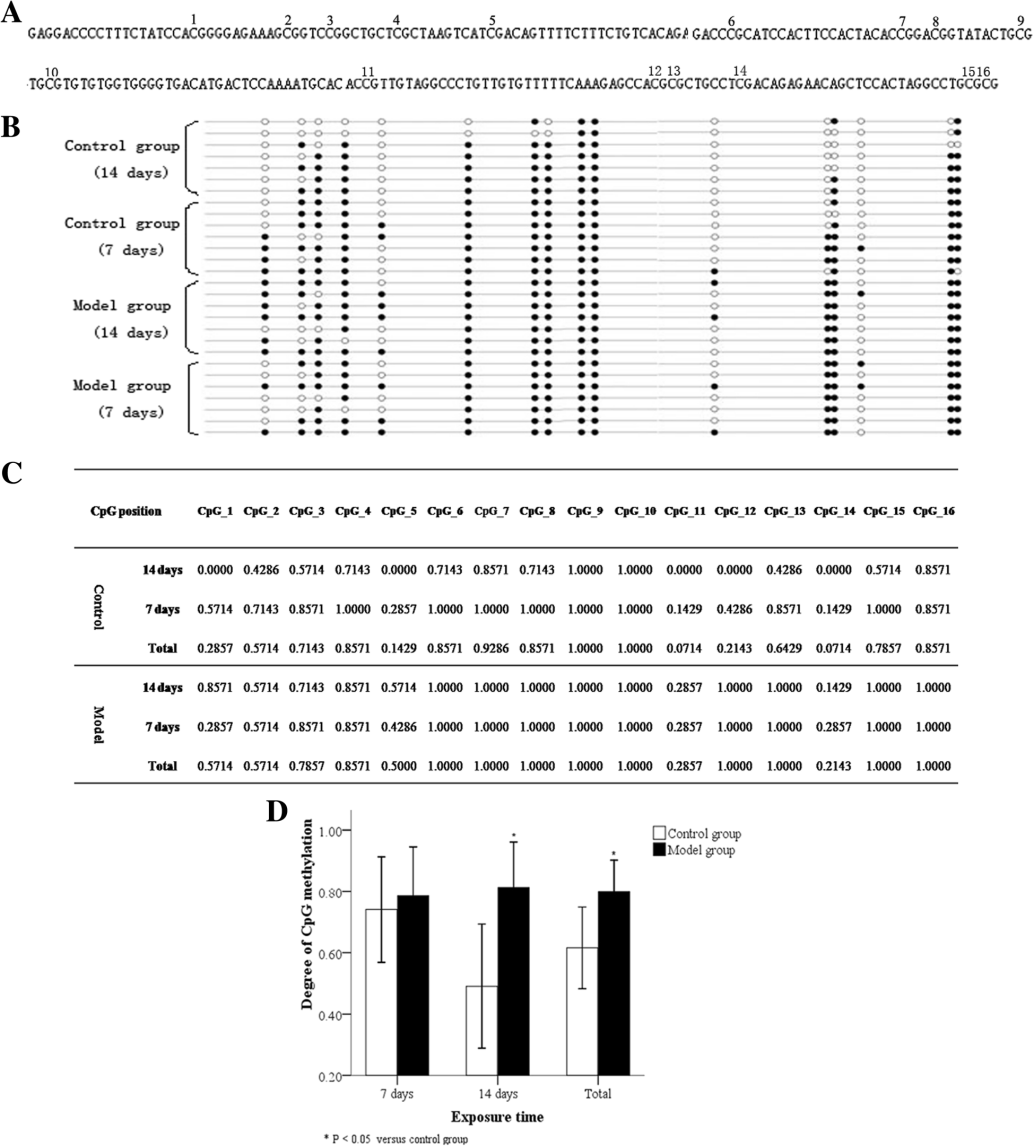
BSP detection of DNA methylation in the *RUNX3* promoter region of AT2 cells. The detection of 16 sites in the *RUNX3* promoter region by BSP demonstrated the DNA methylation percentage of the *RUNX3* promoter region in AT2 cells (**a**). In each specimen, the DNA methylation proportion differed at different sites (**b**, **c**). The DNA methylation proportion was higher in AT2 cells of the model group compared to that in the control group (**d**). Closed circles: methylated CpGs; open circles: unmethylated CpGs. *P < 0.05 vs. control group, n = 7

We also examined the promoter methylation of *RUNX3* in AT2 cells. AT2 cells in both the control and model groups were found to be in a high methylation state, with the methylation proportion in the model group significantly higher (Fig. 7; *P* < 0.05). However, compared with the control group, the DNA methylation proportion in the model group was not significantly different at 7 days, but a high methylation state was observed by 14 days (Fig. 7). Thus the *RUNX3* promoter of AT2 cells derived from the model group under hyperoxia showed hypermethylation compared to that of the control group.

### Binding between H3K27me3/EZH2 and the *RUNX3* promoter

**Fig. 8:**
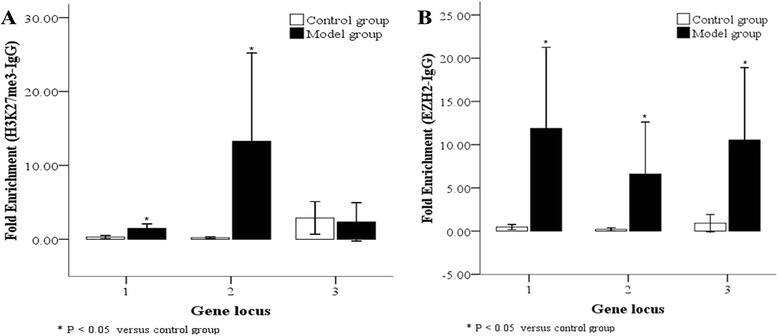
Binding between H3K27me3/EZH2 and the *RUNX3* promoter in AT2 cells. As determined by ChIP, binding levels between H3K27me3 (**a**) or EZH2 (**b**) and the *RUNX3* promoter region were higher in AT2 cells after 14 days of hyperoxia compared to the control group. Gene locus 1: RUNX3 promoter region −5407 ~ −5142 bp; 2: RUNX3 promoter region −5407 ~ −5215 bp; 3: RUNX3 promoter region −5407 ~ −5142 bp. *P < 0.05 vs. control group, n = 10

We also examined binding between H3K27me3 or EZH2 and the *RUNX3* promoter in AT2 cells from control and model groups. Compared with the control group, *RUNX3* sequence 1 (−5407 ~ −5142 bp) and sequence 2 (−5407 ~ −5215 bp) both significantly bound to H3K27me3 or EZH2 at high levels in the BPD model group at 14 days (Fig. 8a, b; *P* < 0.05); binding between *RUNX3* sequence 2 and H3K27me3 was more significant (*P* < 0.05). The binding level of *RUNX3* sequence 3 (−571 ~ −391 bp) with EZH2 was also significantly higher (Fig. 8a, b; *P* < 0.05), but that with H3K27me3 was not significantly different in the BPD model group compared to control. Thus binding occurs between the *RUNX3* promoter and H3K27me3 or EZH2 in AT2 cells of the BPD model group. This suggests EZH2-mediated H3K27 methylation occurs at the *RUNX3* promoter under hyperoxia.

### RUNX3 protein and mRNA expression over time in AT2 cells following hyperoxia

**Fig. 9:**
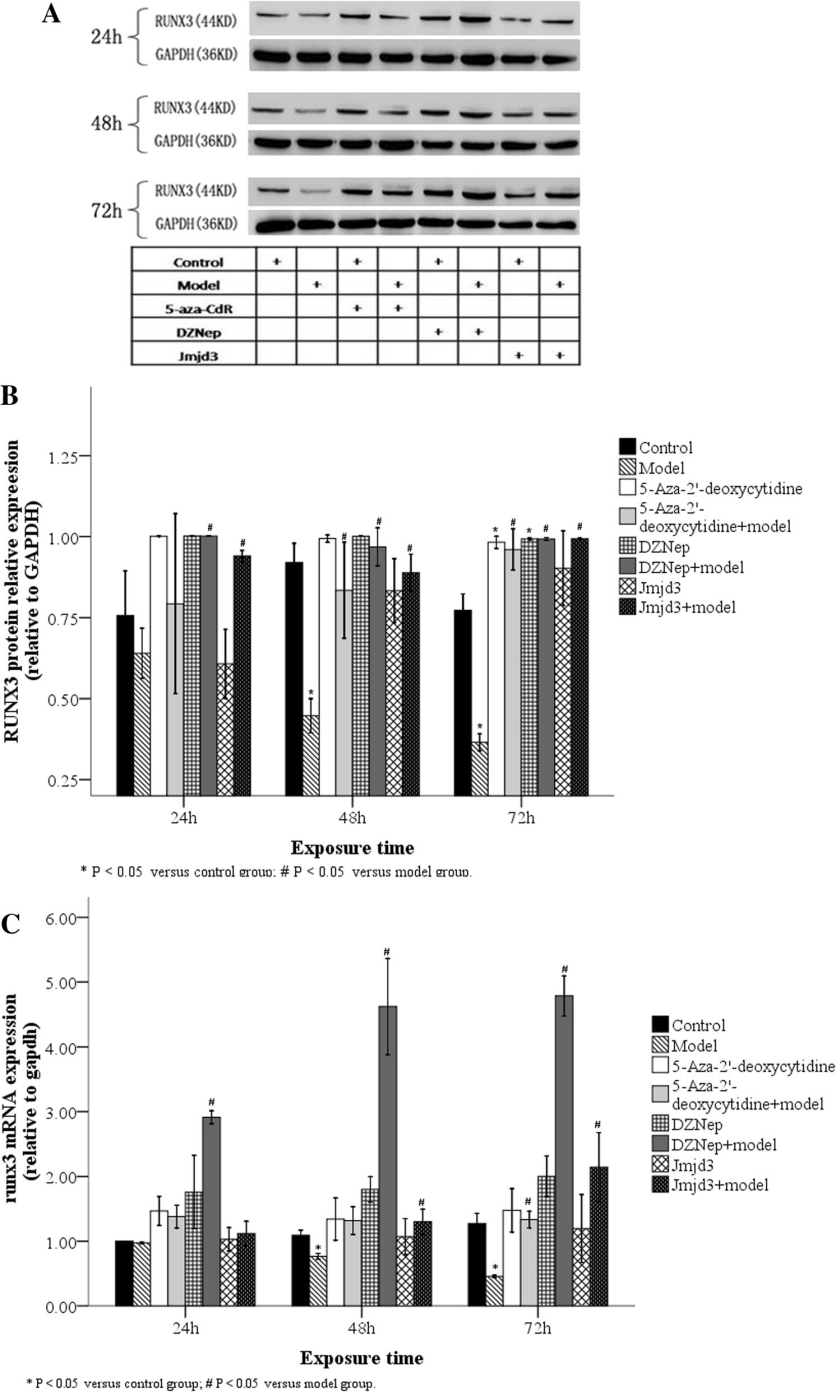
RUNX3 protein and mRNA levels over time in AT2 cells after inhibitor treatment. RUNX3 protein expression in AT2 cells was detected by Western blot at different time points after JMJD3 (20 μg/L), DZNep (1 μmol/L) or 5-Aza-CdR (5 μmol/L) treatment (**a**). In the model group, RUNX3 protein in AT2 cells was up-regulated after 48 h with JMJD3 or DZNep, but not 5-Aza-CdR, treatment (**b**). As detected by real-time PCR, RUNX3 mRNA in AT2 cells of the model group was up-regulated after 72 h treatment with JMJD3, DZNep or 5-Aza-Cd (**c**). 24, 48, 72 h: 24, 48 and 72 h after inhibitor treatment. *P < 0.05 vs. control group; #*P* < 0.05 vs. model group, n = 4

We analyzed the expression of RUNX3 protein and mRNA over time in AT2 cells derived from control and model cells. Compared with the control group, RUNX3 protein and mRNA expression in AT2 cells from the model group cultured in an 85 % O_2_ environment significantly decreased by 48–72 h (Fig. 9a; *P* < 0.05). These results suggest that RUNX3 expression is inhibited by hyperoxia.

### Effects of inhibitor treatment on RUNX3 protein and mRNA expression in AT2 cells

We next analyzed the effects of the inhibition of methylation on RUNX3 protein and mRNA levels in AT2 cells derived from control and model cells. When compared with the control group, RUNX3 protein levels in AT2 cells from the model group were significantly up-regulated at 48 h after treatment with JMJD3, a H3K27 demethylase, or DZNep, an EZH2 inhibitor, but not with the DNA methylation inhibitor, 5-Aza-CdR (Fig. 9b; *P* < 0.05). However, RUNX3 protein and mRNA levels were markedly, and significantly, up-regulated after 72 h treatment with all three inhibitors (Fig. 9b, c; *P* < 0.05). These results suggest that DNMT-mediated DNA methylation and H3K27me3 both induce RUNX3 protein down-regulation after 72 h.

**Fig. 10:**
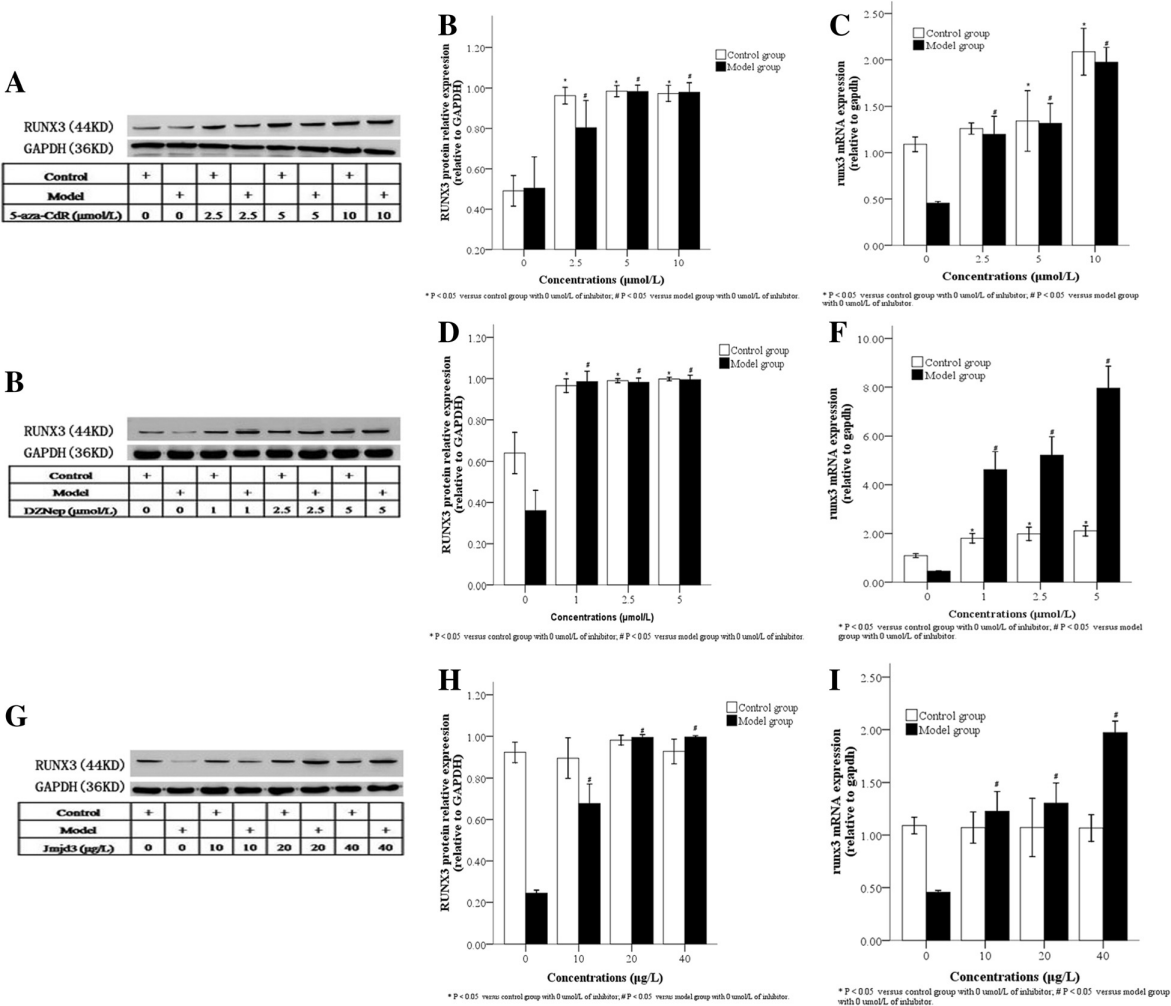
RUNX3 protein and mRNA levels in AT2 cells after 48 h treatment with different concentrations of inhibitors. Cells from control and model groups were treated with various inhibitors. As detected by Western blot, RUNX3 protein expression in AT2 cells from both control and model groups increased after 48 h treatment with different concentrations of 5-Aza-CdR (**a** and **b**, 2.5, 5, 10 μmol/L), DZNep (**d** and **e**, 1, 2.5, 5 μmol/L) or JMJD3 (**g** and **h**, 10, 20, 40 μg/L). As detected by real-time PCR, RUNX3 mRNA expression in AT2 cells from both control and model groups increased after 48 h treatment with different concentrations of 5-Aza-CdR (**c**), DZNep (**f**) or Jmjd3 (**i**). *P < 0.05 vs. control group; #*P* < 0.05 vs. model group, n = 4

AT2 cells were then treated with different concentrations of inhibitors for 48 h. Compared with untreated cells, 2.5 μmol/L 5-Aza-CdR (Fig. 10a, b, c) and 1 μmol/L DZNep (Fig. 10d, e, f) both significantly up-regulated RUNX3 protein and mRNA in AT2 cells from the control group (*P* < 0.05 for both), while 10 μg/L JMJD3 caused no change (Fig. 10g, h, i). Compared with untreated cells, 2.5 μmol/L 5-Aza-CdR (Fig. 10a, b, c), 1 μmol/L DZNep (Fig. 10d, e, f) and 10 μg/L JMJD3 (Fig. 10g, h, i) all significantly up-regulated RUNX3 protein and mRNA in AT2 cells from the model group (Fig. 10; *P* < 0.05). These results suggest that the inhibition of histone or DNA methylation upregulates RUNX3 expression in a dose-dependent manner.

## Discussion

BPD is an infantile disease, frequently seen in premature infants of low fetal age and birth weight, with a complicated pathogenesis. We have previously found that newborn BPD rat lung tissues displayed enlarged alveolar spaces, thickened pulmonary septa, increased alveoli number and secondary septa, a simplified alveolar structure, and a persistently low RAC (a pulmonary development evaluation index), all which indicate a pulmonary developmental disorder [4, 26].

*RUNX3*, a member of the RUNX family, is a gene associated with pulmonary development. At E17.5, RUNX3 protein was widely distributed in bronchioles and alveolar epithelial cells of mouse lung tissues, and participated in the differentiation of pulmonary epithelial cells [8] and the formation of pulmonary blood vessels [7]. *RUNX3* knock-out mice displayed lethal, excessive proliferation and abnormal differentiation of cells, 24 h after birth [8]. In this study, we found RUNX3 expression in alveolar epithelial cells that was significantly decreased in newborn rats and AT2 cells, 7 days and 48 h, respectively, after hyperoxia induction; noted pathological changes were similar to those in *RUNX3* knock-out mice. This suggests that RUNX3 down-regulation in the late stages of hyperoxia may cause excessive proliferation and abnormal differentiation of AT2 cells, and thus contribute to the occurrence of pulmonary developmental disorder in BPD.

In recent years, numerous studies have suggested that BPD is a genetically susceptible disease, to which both environment and genetics jointly contribute [1]. For instance, the BPD status of one twin was found to be a significant predictor of BPD in the second twin, with a higher incidence noted in homozygotic twins [18, 19]. Epigenetics is a post-transcriptional or post-translational modification mechanism that does not require a change of gene sequence or genetic transmission to the next generation by cell division [28]; an example is the intrauterine or postpartum environment causing DNA methylation, making infants susceptible to chronic obstructive pulmonary disease, asthma and other respiratory system diseases [9, 29]. Does hyperoxia-induced BPD also involve an epigenetic mechanism, notably, commonly occurring DNA or histone methylation? In this study, we found the increased expression of the DNA methylation modification enzymes, DNMT1 and DNMT3b, and the H3K27me3 modification enzyme, EZH2, in lung tissues of the BPD model group from 7 and 10 day, respectively, with similar results in AT2 cells. These findings indicate that DNA methylation and H3K27me3 modification may exist in BPD.

Many studies have reported that *RUNX3* is a CpG island methylation phenotype, rich in CG sites [29]. *RUNX3* often regulates expression at the post-transcriptional level by DNA methylation between the 5′-terminal and TSS of genes [9]. Considering the possible presence of DNA methylation in BPD, we hypothesized that the down-regulated expression of pulmonary development-related genes such as *RUNX3* in BPD could be associated with such a modification mechanism. We therefore analyzed correlations between RUNX3 protein and DNMT expression in AT2 cells. DNMT1 protein was only highly expressed after 1 day of hyperoxia, while DNMT3b protein was highly expressed at a significant level from 10 day of hyperoxia onwards, and markedly and negatively correlated with RUNX3 protein. We therefore inferred that RUNX3 down-regulation in AT2 cells may be related to DNA methylation mechanisms in BPD.

DNA methylation involves the transfer of a methyl group from S-adenosylmethionine to cytosine on two CG nucleotides of DNA to form 5-methylcytosine; DNMTs catalyze this reaction, which leads to the silencing of gene expression [15, 16]. Using BSP, we found that in both the model and control groups, the *RUNX3* promoter in AT2 cells demonstrated a high percentage of DNA methylation after between 7–14 days of hyperoxia, with a greater proportion in the model group after 14 days of hyperoxia. We therefore speculated that DNA methylation may contribute to RUNX3 down-regulation in the late stages of BPD. Although we unexpectedly found a high incidence of methylation in the control group, especially 7 days after hyperoxia, this incidence was lower than that in the model group, with no significant difference between the two groups.

Most tumor studies show a low probability of *RUNX3* DNA methylation in normal tissue specimens [30], so the observation that *RUNX3* in newborn rat lung tissues displayed differential DNA methylation at different intervals after birth is puzzling. Scholars have found that a very low concentration of oxygen can change DNMT activity in DNA injury [31]. In the early stages after birth, newborn rats are transferred to a postpartum hyperoxic environment from an intrauterine hypoxic environment [32], so their lung tissues are stimulated by relative hyperoxia. We postulated that DNA methylation in the control group was attributable to a change in DNMT activity caused by the transient stimulation of relative hyperoxia after birth; however this hypothesis has yet to be tested.

Common DNMTs include DNMT1, which maintains and regulates DNA methylation, and DNMT3a/b, which establishes *de novo* methylation [15, 16]. It was thought that the major contributor of *RUNX3* DNA methylation was DNMT1; however when DNMT1 siRNA inhibited target cells, RUNX3 expression partially recovered, which was not observed after DNMT3b inhibition [17]. DNMT3b is thought to be a key enzyme in the regulation of gene methylation. Deng et al. [10] treated a colorectal cancer cell line with 5-Aza-CdR and found that DNMT3b, but not DNMT1, expression was inhibited, causing upregulated RUNX3 expression. Similarly, in this study, we observed DNMT3b expression in AT2 cells was negatively related to RUNX3 protein expression. RUNX3 expression in AT2 cells was up-regulated after inhibition using different concentrations of 5-Aza-CdR over time. We therefore inferred that DNMT3b played a dominant role in the DNA methylation of the *RUNX3* promoter.

However, others have proposed that the silencing of RUNX3 expression is associated with EZH2-mediated H3K27me3 modification [10]. This involves the amino terminal of the histone binding to a specific modification group under the action of the methyltransferase, EZH2 [11], and demethyltransferase, JMJD3/UTX [12], to reduce transcription [13]. Hence, H3K27me3 is a stable, inhibitory chromatin marker. H3K27me3 protects and maintains cell totipotency by inhibiting the expression of regulatory genes in embryonic stem cells [33]. AT2 cells are similar to stem cells, and can differentiate into AT1 cells in the late stages of embryonic development [34], and into myofibroblasts in the late stages of epithelial-mesenchymal transition (EMT) [35]. This study showed that, in either lung tissues or extracted AT2 cells, the expression of EZH2 in the BPD model group was significantly higher than that in the control group, and was negatively correlated to RUNX3 protein. Therefore, we postulated that in the intrauterine development phase and the postpartum, abnormal differentiation process of BPD, RUNX3 down-regulation may be related to EZH2-mediated H3K27me3 modification.

Fujii et al. [14] found that *EZH2* knock-out reduced H3K27me3-binding RUNX3 levels and thus up-regulated *RUNX3* mRNA levels; they believed *RUNX3* silencing was a result of EZH2-dependent H3K27me3 modification. In this study, using a ChIP technique, we detected H3K27me3-binding and EZH2-binding RUNX3 levels in AT2 cells extracted from lung tissues of the model and control groups at 14 days, with higher levels found in the model group. The −5407 ~ −5215 sequence in the promoter region tightly bound to H3K27me3, while the sequence between −5407 ~ −5142 and −571 ~ −391 tightly bound to EZH2. We therefore believe EZH2-mediated H3K27 trimethylation may also be involved in RUNX3 down-regulation in our BPD model.

We found that the downregulation of RUNX3 expression in the late stages of BPD may be due to DNA methylation and H3K27me3 modification. In order to verify this, we treated AT2 cells with inhibitors over time: d5-Aza-CdR, a DNMT inhibitor [36]; JMJD3, an H3K27me3 demethyltransferase that antagonizes EZH2 [37]; and DZNep, which inhibits EZH2 [38]. We detected RUNX3 expression in AT2 cells after inhibitor treatment by Western blot and real-time PCR. We found that by 48 h, 5-Aza-CdR, Jmjd3 and DZNep significantly up-regulated RUNX3 protein and mRNA which suggests that DNMT3b-mediated DNA methylation and H3K27me3 both participate in RUNX3 protein down-regulation during the late stages of hyperoxia-induced BPD.

How do DNA or histone methylation play dominant roles in RUNX3 down-regulation? Researchers found *EZH2* knock-out increased *RUNX3* mRNA levels 3.5–10.4-fold, an effect markedly stronger than that of DNA methylation inhibitors; they therefore inferred that histone modification was a dominant mechanism [14]. However, Liudmila et al. [39] questioned this conclusion, after observing that *EZH2* knock-out alone did not recover gene expression, and that RUNX3 expression recovered after pre-treatment with DNA methylation inhibitors. We found that compared with the model group at the same time points, RUNX3 protein and mRNA levels in the control group were both significantly up-regulated at 48 h after treatment with the histone methylation inhibitor, JMJD3, or EZH2 inhibitor, DZNep; the effect of the DNA methylation inhibitor, 5-Aza-CdR, was less significant, with a change only observed after 72 h. Therefore, we believe that during RUNX3 protein down-regulation in the late stages of hyperoxia-induced BPD, EZH2-mediated H3K27me3 plays a dominant role to that of DNMT3b-mediated methylation.

## Conclusions

In conclusion, this study confirms not only the co-presence of two epigenetic modification mechanisms–DNA methylation and H3K27me3–in a newborn rat model of hyperoxia-induced BPD, but also the joint contribution of DNMT3b-mediated DNA methylation and EZH2-mediated H3K27me3 to RUNX3 protein down-regulation in the late stages of BPD. This suggests that, by early screening and treatment with epigenetic modification mechanisms, we can hope to minimize damage induced by environmental and genetic factors to premature infants and the risk of lung diseases like BPD occurring in the future.

## Abbreviations

BPD: Bronchopulmonary dysphasia
DNMT1: DNA methyltransferase 1
DNMT3b: DNA methyltransferase 3b
H3K27me3: Trimethylated lysine 27 on histone H3
EZH2: Enhancer of Zeste Homolog 2
RUNX3: Runt-related transcription factor 3
BSP: Bisulfite sequencing PCR
ChIP: Chromatin Immunoprecipitation Assay
AT2 cells: Alveolar type 2 epithelial cells
EMT: Epithelial-Mesenchymal Transition
5-Aza-CdR: 5-Aza-2′-deoxycytidine
JMJD3: jumonji domain containing 3
DZNep: 3-Deazaneplanocin A
